# Use of population input functions for reduced scan duration whole-body Patlak ^18^F-FDG PET imaging

**DOI:** 10.1186/s40658-021-00357-8

**Published:** 2021-02-05

**Authors:** Joyce van Sluis, Maqsood Yaqub, Adrienne H. Brouwers, Rudi A. J. O. Dierckx, Walter Noordzij, Ronald Boellaard

**Affiliations:** 1 Department of Nuclear Medicine and Molecular Imaging, University Medical Center Groningen, University of Groningen, Hanzeplein 1, 9713 GZ Groningen, The Netherlands; 2 Department of Radiology and Nuclear Medicine, Cancer Center Amsterdam, Amsterdam UMC, Vrije Universiteit Amsterdam, Amsterdam, The Netherlands

**Keywords:** Patlak, PET/CT, Dynamic imaging, Population input function, Scan time

## Abstract

**Abstract:**

Whole-body Patlak images can be obtained from an acquisition of first 6 min of dynamic imaging over the heart to obtain the arterial input function (IF), followed by multiple whole-body sweeps up to 60 min pi. The use of a population-averaged IF (PIF) could exclude the first dynamic scan and minimize whole-body sweeps to 30–60 min pi. Here, the effects of (incorrect) PIFs on the accuracy of the proposed Patlak method were assessed. In addition, the extent of mitigating these biases through rescaling of the PIF to image-derived IF values at 30–60 min pi was evaluated.

**Methods:**

Using a representative IF and rate constants from the literature, various tumour time-activity curves (TACs) were simulated. Variations included multiplication of the IF with a positive and negative gradual linear bias over 60 min of 5, 10, 15, 20, and 25% (generating TACs using an IF different from the PIF); use of rate constants (*K*_1_, *k*_3_, and both *K*_1_ and *k*_2_) multiplied by 2, 1.5, and 0.75; and addition of noise (*μ* = 0 and *σ* = 5, 10 and 15%). Subsequent Patlak analysis using the original IF (representing the PIF) was used to obtain the influx constant (*K*_*i*_) for the differently simulated TACs. Next, the PIF was scaled towards the (simulated) IF value using the 30–60-min pi time interval, simulating scaling of the PIF to image-derived values. Influence of variabilities in IF and rate constants, and rescaling the PIF on bias in *K*_*i*_ was evaluated.

**Results:**

Percentage bias in *K*_*i*_ observed using simulated modified IFs varied from − 16 to 16% depending on the simulated amplitude and direction of the IF modifications. Subsequent scaling of the PIF reduced these *K*_*i*_ biases in most cases (287 out of 290) to < 5%.

**Conclusions:**

Simulations suggest that scaling of a (possibly incorrect) PIF to IF values seen in whole-body dynamic imaging from 30 to 60 min pi can provide accurate *Ki* estimates. Consequently, dynamic Patlak imaging protocols may be performed for 30–60 min pi making whole-body Patlak imaging clinically feasible.

## Introduction

Positron emission tomography integrated with computed tomography (PET/CT) imaging using ^18^F-2-fluoro-2-deoxy-d-glucose (^18^F-FDG) is widely used in oncology for diagnosis, staging, and treatment response evaluation [1–11]. The standardized uptake value (SUV), a semi-quantitative metric derived from PET images, is most commonly used as a surrogate of metabolic activity for quantifying ^18^F-FDG tumour uptake [1]. SUV can be derived from static PET acquisitions, typically initiated 1 h post-injection (pi) where every bed position is scanned once for 2–5 min [1, 12]. Through standardization methods regarding patient preparation (to avoid, e.g., high plasma glucose), PET acquisition settings, image reconstruction, and analysis methods, SUV variability can be limited to a great extent [1, 13, 14]. However, quantitative accuracy of SUV can also be influenced by changes in plasma kinetics due to treatment possibly causing inaccurate assessments [15–17].

Dynamic PET imaging allows for spatiotemporal activity concentration distribution measurement which can provide voxel-wise metabolic information when used by tracer pharmacokinetic modelling methods, for example, full kinetic analysis, i.e., Patlak analysis [18–21].

Up to recently, dynamic PET imaging was mainly performed using single-bed/single-axial field-of-view acquisitions. Currently, with state-of-the-art PET/CT systems, whole-body dynamic (Patlak) images can be obtained from a combined acquisition of first 6 min of dynamic imaging over the heart to obtain the arterial input function (IF) followed by multiple whole-body sweeps up to 60 min pi. This procedure followed by a standard static whole-body PET, however, can take a total examination time to 75 min [22], including the time needed for patient positioning and CT procedure. Initial PET examinations using whole-body Patlak imaging showed a high frequency of patients’ inability to comply with the long scan duration required for the protocol.

The use of a population-averaged input function (PIFs) could obviate the need for the first dynamic scan and minimize whole-body sweeps to an interval of 30–60 min pi [23] making whole-body dynamic Patlak imaging clinically feasible. There have been various studies in which using a PIF is explored in oncological whole-body dynamic ^18^F-FDG imaging [21, 23–26]. Promising results were obtained in comparison with using an arterial IF and an image derived IF; however, further evaluation of microparameter estimation (such as *k*_3_ and (if it exists) *k*_4_) is recommended before implementation [24]. Therefore, given that this approach may introduce some inaccuracy, this study explored the effects of (incorrect) PIFs on the accuracy of the proposed Patlak method using various simulations including variations in rate constants. In addition, the extent of mitigating these biases through rescaling of the PIF to image-derived values at 30–60 min pi was evaluated.

## Materials and methods

To explore the effects of (possibly incorrect) PIFs on the accuracy of Patlak analysis based on dynamic whole-body PET acquisition from 30 to 60 min pi, various tumour time-activity curves (TACs) were simulated. To this aim, a representative IF from previously acquired data (acquisition and processing described in [21]) was used as the PIF as well as rate constants based on literature: *K*_1_ was 0.301 min^−1^, *k*_2_ was 0.600 min^−1^, and *k*_3_ was 0.047 min^−1^ [18, 27]. TACs were created according to Eq. 1:

1 $$ {C}_{\mathrm{tissue}}={C}_{\mathrm{blood}}\bigotimes \left[{k}_2{e}^{-\left({k}_2+{k}_3\right)t}+{k}_3\right]\frac{K_1}{k_2+{k}_3} $$

Here, *C*_blood_ represents the original representative left ventricle arterial IF, and *C*_tissue_ is the obtained TAC. The PET TAC was then generated using Eq. 2:

2 $$ {C}_{\mathrm{PET}}=\left(1-{V}_b\right)\times {C}_{\mathrm{tissue}}+{V}_b\times {C}_{\mathrm{blood}} $$

with *V*_*b*_ equal to the blood volume fraction.

Simulations were performed in the Python programming language using an in-house written code.

Through the multiplication of the IF with a positive and negative gradual linear bias using different slopes over 60 min, variations in IF were simulated. Differences in slope steepness included a positive and negative gradual linear bias from one to 5, 10, 15, 20, and 25% multiplied by the IF, e.g., the IF was multiplied by a positive and negative linear function starting at *y* = 1 to *y* = 1.25 and starting at *y* = 1 to *y* = 0.75 in the case of 25% bias. These simulations were also reversed, e.g., the IF was multiplied by a positive and negative linear function starting at *y* = 1.25 to *y* = 1 and starting at *y* = 0.75 to *y* = 1 in the case of 25% bias. Variations in IF accounted for a total number of 35 simulations.

Other variations that were combined with the gradual bias from one to different slope sizes included the use of rate constants (*K*_1_, *k*_3_, and both *K*_1_ and *k*_2_) multiplied by 2, 1.5, and 0.75 (fitting the mean and exceeding the range of rate constants observed in clinical data [27]). These rate constant variations combined with IF modifications accounted for a total number of 180 simulations. In addition, noise was added to the IF modifications (*μ* = 0 and *σ* = 5, 10, and 15% simulating high, medium, and low noise equivalent count rate [28], respectively) to account for the differences between PET/CT systems and reconstruction settings. The noise additions combined with IF modifications accounted for a total number of 75 simulations. For the simulations described above, the blood volume fraction was fixed at 8.9% [27].

**Fig. 1:**
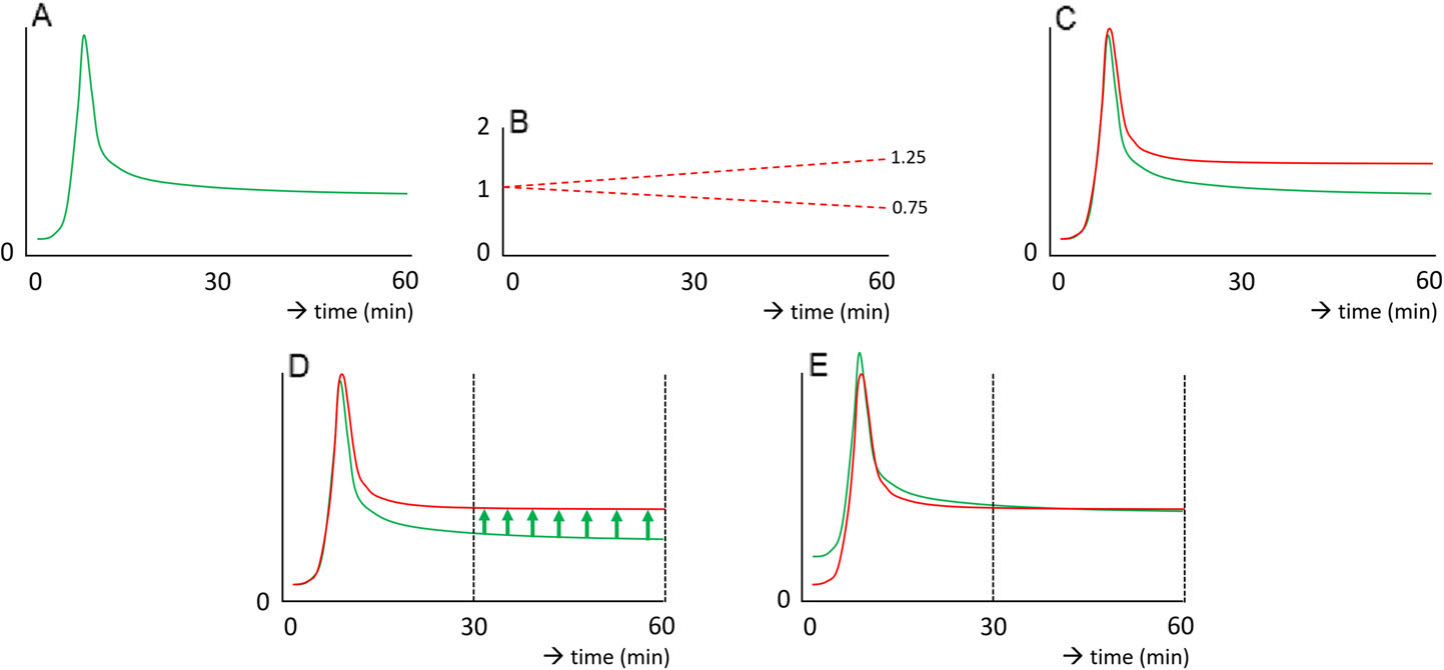
Schematic overview of PIF (green) with a gradual linear modification (dashed red) towards a variety of (incorrect) IFs (red) (**a**–**c**) and subsequent rescaling of the PIF at 30–60 min pi (**d**, **e**)

To show the extent to which incorrect PIFs influence the accuracy of Patlak analysis, analyses using the original IF (representing the PIF) were used to obtain the influx constant (*K*_*i*_) for the differently simulated TACs. Subsequently, the incorrect PIFs were scaled towards the correct value using the 30–60-min pi time-interval simulating scaling of the PIF to image-derived values. After rescaling, *K*_*i*_ were obtained using these adjusted PIFs to see the extent to which previously obtained biases in *K*_*i*_ could be mitigated. A schematic overview regarding the modification and rescaling of the IF is shown in Fig. 1.

## Results

In total, 290 variations were simulated (35 gradual in or decrease of the IF and reversed, 180 rate constant variations, and 75 noise simulations). Percentage bias in *K*_*i*_ using simulated incorrect IFs varied from − 16 to 16% depending on the simulation type, amplitude, addition of noise, and direction of the IF modifications. Subsequent scaling of the PIF reduced these *K*_*i*_ biases in most cases to between − 3 and 4% for the gradual in and decrease of the IF and reversed, between − 3.7 and 4% for the rate constant variations combined with gradual in and decrease of the IF, and between − 2.4 and 3% for the noise addition combined with gradual in and decrease of the IF.

**Fig. 2:**
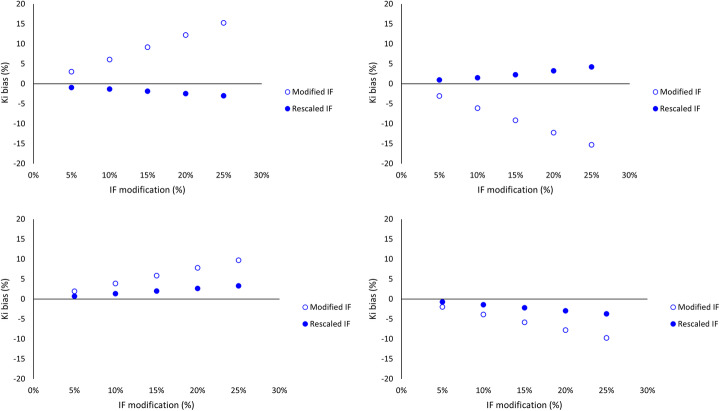
The effect of positive (**a**), negative (**b**), positive reversed (**c**), and negative reversed (**d**) gradual input function modification over 60 min of 5, 10, 15, 20, and 25% on the percentage difference in *K*_*i*_. The empty markers represent the effect of the modification on *K*_*i*_. The filled markers show the remainder of this bias after rescaling of the PIF

**Fig. 3:**
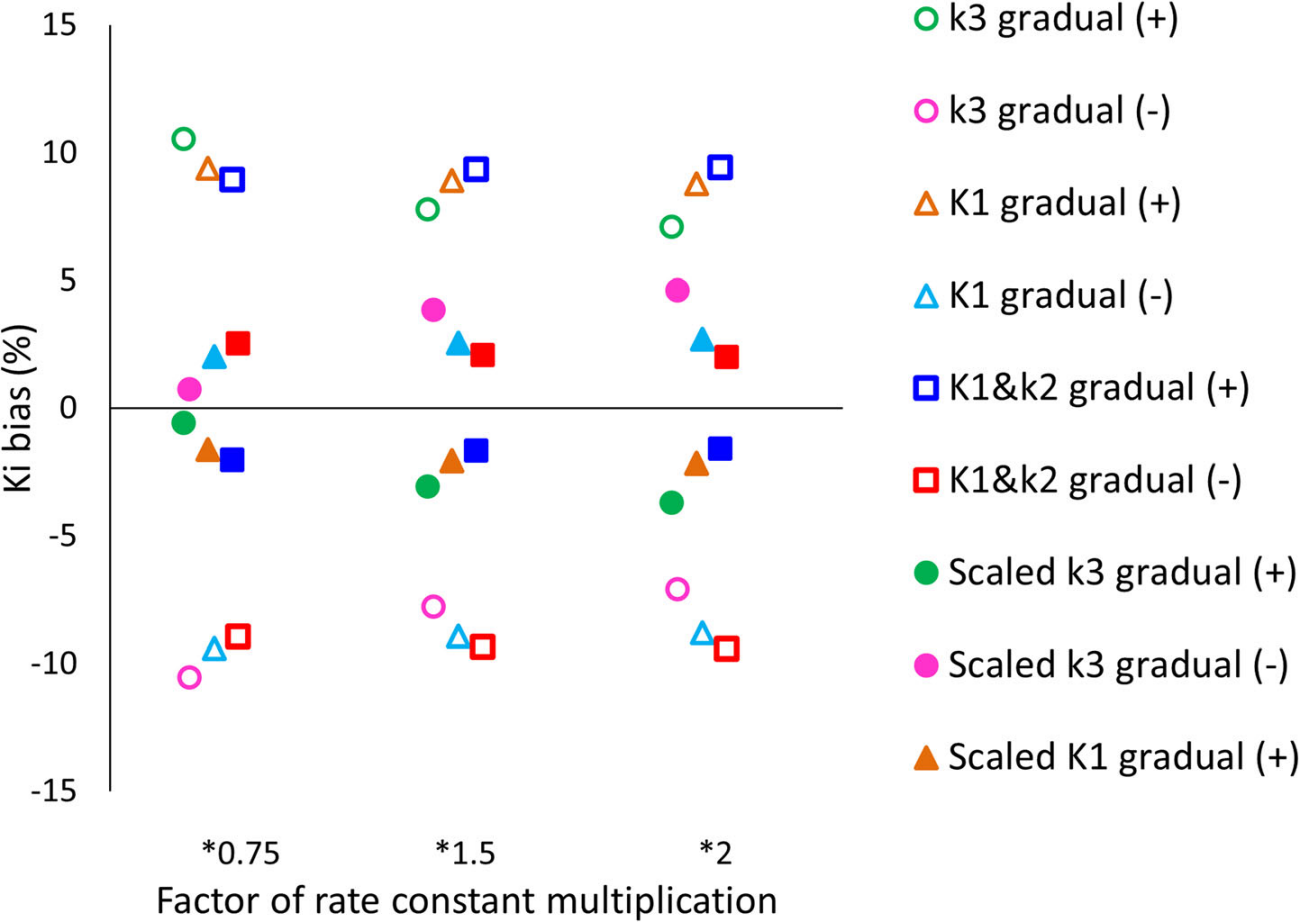
The effect of variations in rate constants combined with 15% gradual modification of the IF on the percentage difference in *K*_*i*_. The empty markers represent the effect of the modification on *K*_*i*_. The filled markers show the remainder of this bias after rescaling of the PIF

**Fig. 4:**
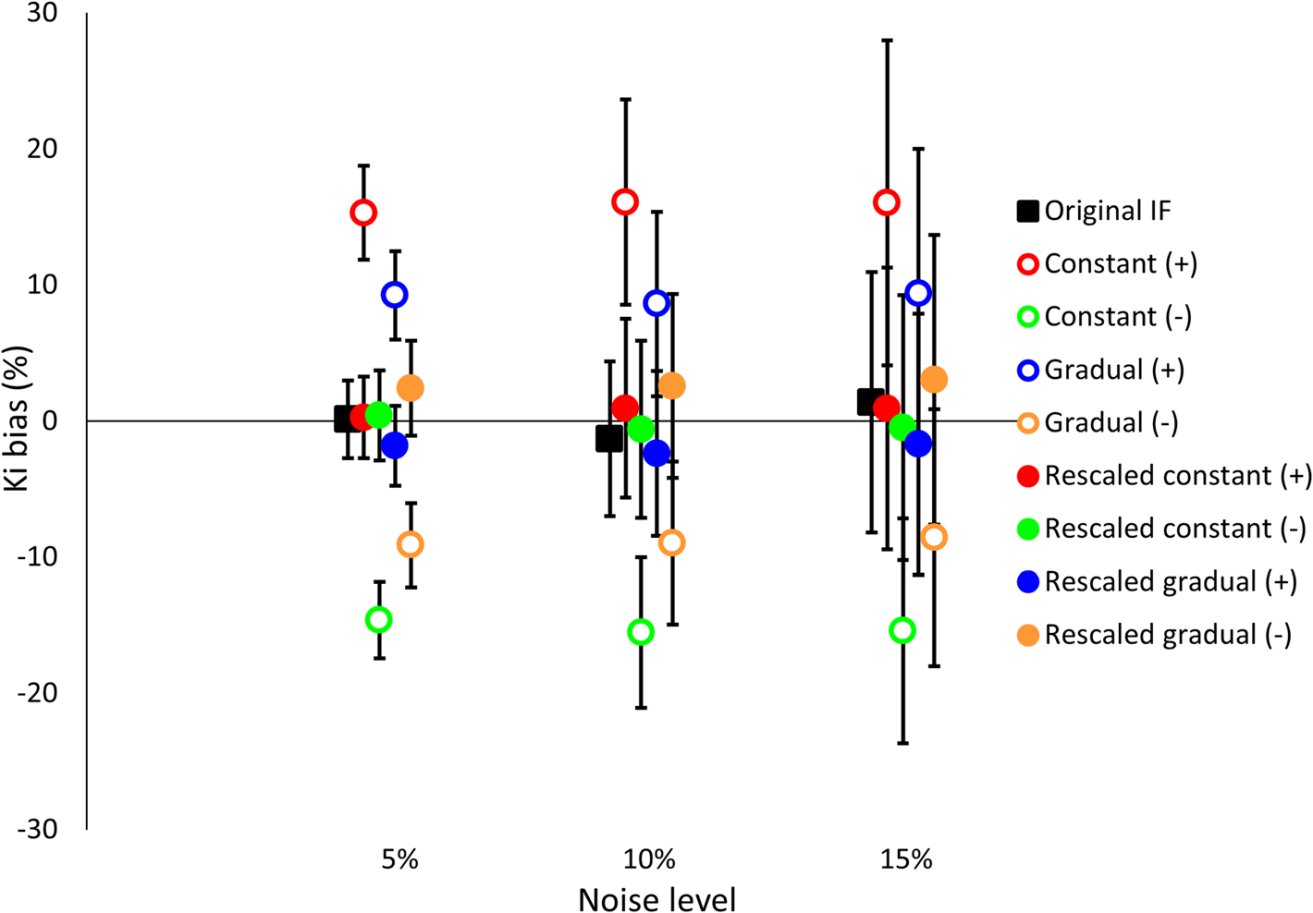
The effect of added noise combined with 15% gradual modification of the IF on the percentage difference in *K*_*i*_. The original input function with added noise is represented by the filled black marker. The empty markers represent the effect of the modification on *K*_*i*_. The filled markers show the remainder of this bias after rescaling of the PIF

Figure 2 shows the influence of positive, negative, and reversed gradual input function modification over 60 min of 5, 10, 15, 20, and 25% on the percentage difference in *K*_*i*_ before and after rescaling of the PIF. The effect of variations in rate constants combined with a gradual modification of the IF on the percentage difference in *K*_*i*_ before and after rescaling of the PIF is shown in Fig. 3. For the influence of added noise combined with a gradual modification of the IF on the percentage difference in *K*_*i*_ before and after rescaling of the PIF, see Fig. 4.

## Discussion

Tumour quantification using SUVpeak following the PERCIST guidelines describes stable metabolic disease as an increase or decrease in SUVpeak of less than 30% [29]. However, SUV can be affected by variations (between subjects or longitudinally) of the plasma kinetics which may cause inaccuracies in clinical assessments. Nevertheless, Patlak analysis also remains an estimate of the tumour’s kinetic behaviour and may be biassed in case of large blood volume fractions for example in cases with bulky tumours [21]; this should be taken into account. Based on the reasoning as described above, we determined that an accuracy level of 5% is acceptable, i.e., well within repeatability levels (which are in the order of 10 to 15% [30–32]).

In most cases, rescaling of the PIF reduced *K*_*i*_ biases to < 5%. However, there were three incidences (out of 290) which resulted in remaining biases > 5% after rescaling: the combination of 25% gradual IF modification and *k*_3_*2 resulted in a remaining *K*_*i*_ bias of 8%, the combination of 25% gradual IF modification and *k*_3_*1.5 resulted in a remaining *K*_*i*_ bias of 7%, and the combination of 20% gradual IF modification and *k*_3_*2 resulted in a remaining bias in *K*_*i*_ of 6%. When using PIFs for whole-body Patlak imaging in patients with high nuclear grade and/or high proliferation activity tumours (associated with higher *k*_3_ [33]), this should be taken into account.

Please note that when using Patlak analysis, bias may occur even with a perfectly correct IF. Bias in the Patlak analysis may occur as a result of not incorporating the fractional blood volume in the Patlak equations. Blood volume fractions of 0, 8.9, and 20% resulted in a Patlak *K*_*i*_ bias of − 1.2, − 10, and − 20%, respectively (data not shown). This indicates that the *K*_*i*_ bias through the use of a (possibly incorrect, but rescaled) PIF is small compared to possible biases in case of large blood volume fractions. With regard to the acceptable accuracy level of 5% when using a PIF for estimating the tumour kinetic behaviour, the acceptable total bias would be 25%, which is just within the PERCIST recommendations [29]. Yet, for most tumours, the blood volume fraction is typically smaller than 10% [27] resulting in a total acceptable bias of 15%, so again, well within the limits of agreement regarding repeatability and PERCIST criteria.

A similar study to develop a simplified Patlak protocol through using PIFs based on clinical data was performed by S. Yao et al. [26]. Here, similar biases (from − 20 to 20%) were induced to a representative IF to simulate and explore the effect of possible errors in PIFs when applied at 20 min pi. They concluded that whenever the IF modification remains below 20%, quantitative inaccuracy regarding *K*_*i*_ would be around 4% [26], which is in line with the results of our simulations.

Another study that explored the alternative of using a PIF instead of arterial blood sampling found a very high correlation between the two methods [23]. The addition of variability in rate constants and noise in our study provides a more comprehensive and realistic reflection of the possible range in kinetic parameters seen in tumours.

## Conclusion

Simulations suggest that scaling of a possibly incorrect PIF to (image derived) IF values seen in whole-body dynamic imaging from 30 to 60 min pi could be a good strategy to obtain accurate *K*_*i*_ estimates. Consequently, dynamic Patlak imaging protocols may be performed for 30–60 min pi making whole-body Patlak imaging clinically feasible.

## Data Availability

All data generated during the current study are available from the corresponding author on reasonable request.
